# Assessment of serum biomarkers in patients with plaque psoriasis on secukinumab

**DOI:** 10.1111/1346-8138.15278

**Published:** 2020-03-15

**Authors:** Akimichi Morita, Yumiko Tani, Kazuko Matsumoto, Masako Yamaguchi, Rie Teshima, Mamitaro Ohtsuki

**Affiliations:** ^1^ Department of Geriatric and Environmental Dermatology Nagoya City University Graduate School of Medical Sciences Nagoya Japan; ^2^ Novartis Pharma K.K. Tokyo Japan; ^3^ Department of Dermatology Jichi Medical University Shimotsuke Japan

**Keywords:** biomarker, interleukin‐17A, psoriasis, secukinumab, β‐defensin 2

## Abstract

The molecular basis of interleukin (IL)‐17A in driving psoriasis pathogenesis is not fully elucidated yet. To investigate the underlying mechanisms and biomarkers associated with IL‐17A and the role in psoriasis pathogenesis, over 30 serum proteins were evaluated in a study assessing the effectiveness and safety of secukinumab, where treatment was directly switched from cyclosporin A to secukinumab. Serum β‐defensin 2 (BD‐2) levels rapidly and robustly reduced following secukinumab treatment. BD‐2 levels were well‐correlated with Psoriasis Area and Severity Index (PASI) score; changes in BD‐2 levels preceded change in PASI score. Serum BD‐2, an easily measurable protein, can possibly be used as a suitable surrogate biomarker to monitor responses to IL‐17A‐targeted therapies for psoriasis in clinical practice.

## Introduction

The rapid and robust improvement in psoriatic symptoms with interleukin (IL)‐17A‐targeting therapies including secukinumab, a fully human monoclonal anti‐IL‐17A antibody, enhanced the understanding of the pathogenesis of psoriasis (PsO).^1^, ^2^, ^3^, ^4^, ^5^ Evaluating gene expression in skin biopsy specimens from patients with PsO revealed a set of genes related to PsO, the expression of which changed following IL‐17A inhibitor therapy.^6^, ^7^ However, studies evaluating serum biomarkers in patients with PsO on IL‐17A inhibitor therapy are scarce.^3^, ^5^, ^8^, ^9^

Biologics are often used after primary or secondary failure with non‐biologic systemic treatment such as cyclosporin A (CyA). However, abrupt cessation of systemic treatment could cause disease exacerbation.^10^ In order to evaluate the safety and effectiveness of secukinumab in patient with PsO whose treatment was directly switched from CyA to secukinumab, a phase 4 study was conducted in Japan.^11^

Most studies evaluating serum biomarkers in patients with PsO on IL‐17A inhibitor therapy analyzed a limited numbers of targeted proteins. In the switching study, serum samples were collected to evaluate potential biomarkers that are associated with PsO and/or treatment. Here, we report the data of over 30 serum proteins and their correlation with PsO disease severity as evaluated by Psoriasis Area and Severity Index (PASI) before and after treatment by secukinumab.

## Methods

### Study design

The detailed study design and inclusion criteria have been reported previously.^11^ In short, this is a multicenter, single‐arm, 16‐week, open‐label phase 4 study conducted at 11 sites in Japan between June 2015 and May 2016 to assess the effectiveness and safety of secukinumab‐treated patients directly switched from CyA.

Eligible patients were aged 18 years or older and diagnosed with plaque psoriasis at least 6 months before baseline. Patients had to be taking CyA for at least 12 weeks, and should have experienced a primary or secondary inadequate response (PASI, ≥10; Investigator's Global Assessment modified 2011, ≥2 [scale, 0–4]) to the treatment.

The study protocol was approved by the institutional review board of each participating site, and the study was carried out in accordance with the Declaration of Helsinki and Good Clinical Practice guidelines. Eligible patients provided written informed consent.

### Outcomes

In this switching study, serum samples of patients with moderate to severe PsO were collected to evaluate potential biomarkers that are associated with PsO and/or treatment.

Blood samples were collected at baseline and weeks 2 and 16. Serum chemokines (eotaxin, macrophage inflammatory protein [MIP]‐1α, MIP‐1β, eotaxin‐3, thymus and activation‐regulated chemokine [TARC], interferon‐γ‐inducible protein [IP]‐10, IL‐8 [HA], macrophage‐derived chemokine [MDC], monocyte chemoattractant protein [MCP]‐1, MCP‐4) and cytokines (granulocyte macrophage colony‐stimulating factors [GM‐CSF], tumor necrosis factor [TNF]‐α, TNF‐β, vascular endothelial growth factor [VEGF]‐A, interferon [IFN]‐γ, IL‐1α, IL‐1β, IL‐2, IL‐4, IL‐5, IL‐6, IL‐7, IL‐8, IL‐10, IL‐12/IL‐23p40, IL‐12p70, IL‐13, IL‐15, IL‐16, IL‐17A) were measured by electrochemiluminescence assay according to the manufacturer’s instruction (catalog no. K15054G; Meso Scale Discovery, Rockville, MD, USA). β‐Defensin (BD)‐2 was quantified by enzyme‐linked immunosorbent assay (ELISA) (catalog no. 100‐250‐BD‐2; Alpha Diagnostic International, San Antonio, TX, USA).

### Statistical analysis

Summary of statistics for all of the tested biomarker profiles and the mean change from baseline by visit are presented. Descriptive data are presented in median (quartile range). Correlations of PsO disease and the tested biomarkers were assessed by the Pearson correlation coefficient (*r*‐value) using PASI score and biomarker concentration. *P* < 0.05 was considered statistically significant. Statistical analysis was performed using SAS Studio software 9.4 (SAS Institute, Cary, NC, USA).

## Results

### Patient disposition and clinical effectiveness

Patient disposition and baseline characteristics, as well as clinical effectiveness, have been reported previously.^11^ All 34 patients enrolled in this study completed the required 16 weeks of treatment. The mean PASI score at baseline was 15.05. The primary end‐point of 75% improvement in PASI response (PASI‐75) at week 16 was achieved by 82.4% (*n* = 28) of patients receiving secukinumab.

### Serum biomarkers

**Figure 1:**
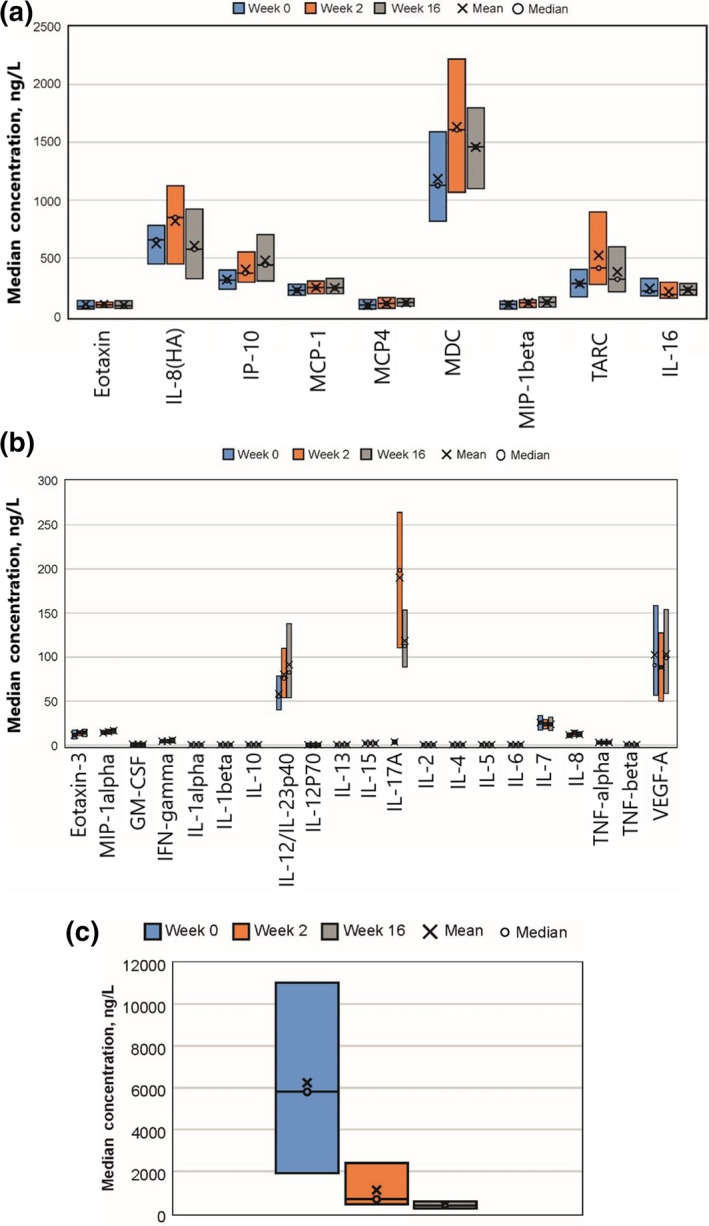
Serum chemokine, cytokine and BD‐2 levels pre‐/post‐secukinumab treatment. Serum (a) chemokine, (b) cytokine and (c) BD‐2 levels were measured in samples collected at baseline, and weeks 2 and 16. Mean (x), median (o), 25 percentile (lower line) and 75 percentile (upper line) are presented. Different detection antibodies were used for IL‐8 measurement in chemokine and cytokine panel according to the manufacturers’ instructions. BD, β‐defensin; GM‐CSF, granulocyte macrophage colony‐stimulating factors; IL, interleukin; IP, interferon‐γ‐inducible protein; MCP, monocyte chemoattractant protein; MDC, macrophage‐derived chemokine; MIP, macrophage inflammatory protein; TARC, thymus and activation‐regulated chemokine; TNF, tumor necrosis factor; VEGF, vascular endothelial growth factor.

Serum protein levels at baseline, namely 1 day after the last dose of CyA and before the first secukinumab dose, are summarized in Table 1. The serum protein changes from baseline by visit are presented in Figure 1. Among the proteins tested, serum BD‐2 level reduced from baseline (median, 5850.00 ng/L [range, 910.00–11 000.00]) to week 2 (729.00 ng/L [451.00–2450.00]), which was sustained at week 16 (407.00 ng/L [257.00–590.00]) (Fig. 1c). A substantial increase in IL‐17A (both free and antibody‐bound forms) was seen from baseline (3.44 ng/L [2.02–5.72]) to week 2 (198.00 ng/L [111.00–264.00]), which was sustained at week 16 (112.00 ng/L [89.60–154.00]) (Fig. 1b).

**Table 1:** Serum proteins at baseline

Chemokine	Median (quartile), ng/L	Reference,^12^ median, pg/mL†	Cytokine	Median (quartile), ng/L	Reference,^13^, ^14^ median, pg/mL†
Eotaxin	98.0 (74.4–142.0)	55.9	GM‐CSF	0.31 (0.24–0.39)	0.18
Eotaxin‐3	10.1 (6.5–16.6)	8.2	IFN‐γ	3.90 (2.98–5.12)	3.77
IL‐8 (HA)	662.0 (452.0–788.0)	575.0	IL‐1α	0.10 (0.03–0.15)	1.18
IP‐10	319.0 (239.0–404.0)	80.9	IL‐1β	0 (0–0.2)	0.16
MCP‐1	226.5 (188.0–274.0)	118.0	IL‐2	0.27 (0.16–0.33)	0.52
MCP‐4	103.5 (74.0–142.0)	44.3	IL‐4	0.06 (0.03–0.07)	ND
MDC	1130.0 (828.0–1590.0)	1350.0	IL‐5	0.07 (0–0.47)	0.28
MIP‐1α	14.8 (13.7–16.4)	37.0	IL‐6	0.79 (0.56–1.40)	0.47
MIP‐1β	106.0 (72.4–137.0)	53.1	IL‐7	25.60 (18.0–32.80)	0.92
TARC	287.5 (178.0–412.0)	29.1	IL‐8	10.85 (8.56–13.40)	9.61
			IL‐10	0.28 (0.20–0.36)	0.20
**Other**	**Median (quartile), ng/L**	**Reference,** 5 **GM,** **pg** **/** **mL** †	IL‐12/IL‐23p40	56.00 (39.80–79.00)	53.30
BD‐2	5850.0 (1910.0–11 000.0)	0.2	IL‐12p70	0.20 (0.11–0.25)	0.29
			IL‐13	0.79 (0.62–1.05)	1.65
			IL‐15	2.22 (1.87–2.58)	1.29
			IL‐16	224.00 (188.00–328.00)	59.90
			IL‐17A	3.44 (2.02–5.72)	0.93
			TNF‐α	2.42 (1.84–2.86)	0.36
			TNF‐β	0.34 (0.24–0.42)	0.15
			VEGF‐A	90.90 (57.40–157.00)	9.62

^†^ Reference values refer to assay kit product insert and published report on healthy human subjects. pg/mL is equivalent to ng/L. Different detection antibodies were used for IL‐8 measurement in chemokine and cytokine panel according to manufacturers’ instructions. BD, β‐defensin; GM, geometric mean; GM‐CSF, granulocyte macrophage colony‐stimulating factors; IFN, interferon; IL, interleukin; IP, interferon‐γ‐inducible protein; MCP, monocyte chemoattractant protein; MDC, macrophage derived chemokine; MIP, macrophage inflammatory protein; ND, non‐detectable; TARC, thymus and activation‐regulated chemokine; TNF, tumor necrosis factor; VEGF, vascular endothelial growth factor.

**Figure 2:**
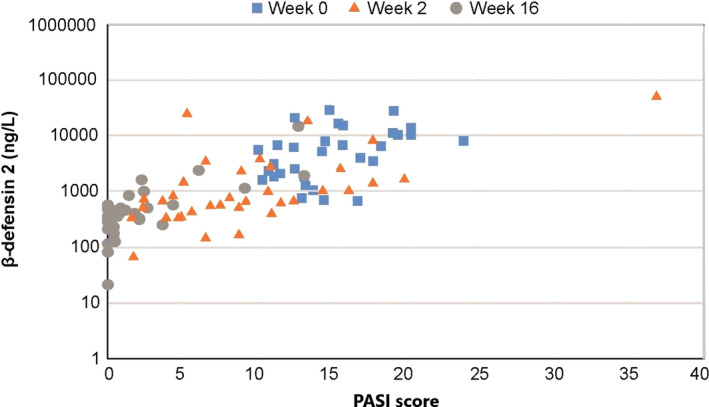
Correlation between PASI score and serum BD‐2 level. Serum samples from baseline, and weeks 2 and 16 were pooled for analysis and the correlation was assessed by Pearson correlation coefficient using PASI score and BD‐2 concentration. Data from FAS. BD, β‐defensin; FAS, full analysis set; PASI, Psoriasis Area and Severity Index.

At baseline, when patients’ disease severity was PASI of more than 10 despite the treatment with CyA, a positive correlation between PASI score and serum concentration was found for MDC (*r* = 0.48, *P* = 0.004), MIP‐1α (*r* = 0.393, *P* = 0.021), IL‐16 (*r* = 0.394, *P* = 0.021) and BD‐2, (*r* = 0.459, *P* = 0.006). When all time points (baseline, week 2, and week 16) were pooled for data analysis, such relationship was lost in MDC, MIP‐1α and IL‐16, whereas BD‐2 showed a stronger correlation (*r* = 0.767, *P* < 0.001) (Table 2, Fig. 2). IFN‐γ levels were inversely correlated with PASI in both baseline (*r* = −0.455, *P* = 0.007) and pooled data analysis (*r* = −0.275, *P* = 0.005), and a negative correlation was observed between serum IL‐17A level and PASI only in the pooled data analysis (*r* = −0.452, *P* < 0.001) (Table 2).

**Table 2:** Correlation between PASI score and serum proteins

	Baseline (*n* = 34)		All time points† (*n* = 102)	
	Correlation coefficient	*P*	Correlation coefficient	*P*
Chemokine				
Eotaxin	0.006	0.972	0.002	0.982
Eotaxin‐3	0.125	0.481	0.022	0.825
IL‐8 (HA)	−0.197	0.263	−0.022	0.83
IP‐10	0.134	0.451	−0.06	0.548
MCP‐1	0.120	0.499	−0.037	0.715
MCP‐4	−0.004	0.983	0.07	0.482
MDC	**0.480**	**0.004**	0.187	0.06
MIP‐1α	**0.393**	**0.021**	0.076	0.448
MIP‐1β	−0.033	0.854	−0.169	0.09
TARC	0.264	0.131	0.167	0.094
Cytokine				
GM‐CSF	−0.173	0.328	−0.117	0.242
IFN‐γ	**−0.455**	**0.007**	**−0.275**	**0.005**
IL‐1α	−0.138	0.435	0.075	0.455
IL‐1β	−0.143	0.419	0.07	0.482
IL‐2	−0.221	0.209	−0.037	0.714
IL‐4	0.256	0.143	0.16	0.109
IL‐5	−0.182	0.303	−0.073	0.464
IL‐6	0.231	0.189	0.167	0.093
IL‐7	0.164	0.354	0.105	0.295
IL‐8	0.122	0.491	0.128	0.199
IL‐10	−0.186	0.293	0.133	0.184
IL‐12/IL‐23p40	0.104	0.557	0.009	0.925
IL‐12p70	−0.247	0.159	−0.164	0.099
IL‐13	0.141	0.428	0.046	0.644
IL‐15	−0.112	0.528	−0.002	0.981
IL‐16	**0.394**	**0.021**	0.195	0.05
IL‐17A	0.220	0.211	**−0.452**	**<0.001**
TNF‐α	0.329	0.058	0.076	0.446
TNF‐β	−0.212	0.228	0.034	0.734
VEGF‐A	0.228	0.194	0.169	0.09
Other				
BD‐2	**0.459**	**0.006**	**0.767**	**<0.001**

^†^ Pearson correlation analysis presented in this table was evaluated using the baseline and pooled data of PASI scores and serum proteins observed at the three time points (baseline, week 2 and week 16). Different detection antibodies were used for IL‐8 measurement in chemokine and cytokine panel according to the manufacturers’ instructions. Bold indicates positive/negative correlation and significance at *P* < 0.05. BD, β‐defensin; GM‐CSF, granulocyte macrophage colony‐stimulating factors; IFN, interferon; IL, interleukin; IP, interferon‐γ‐inducible protein; MCP, monocyte chemoattractant protein; MDC, macrophage‐derived chemokine; MIP, macrophage inflammatory protein; TARC, thymus and activation‐regulated chemokine; TNF, tumor necrosis factor; VEGF, vascular endothelial growth factor.

**Figure 3:**
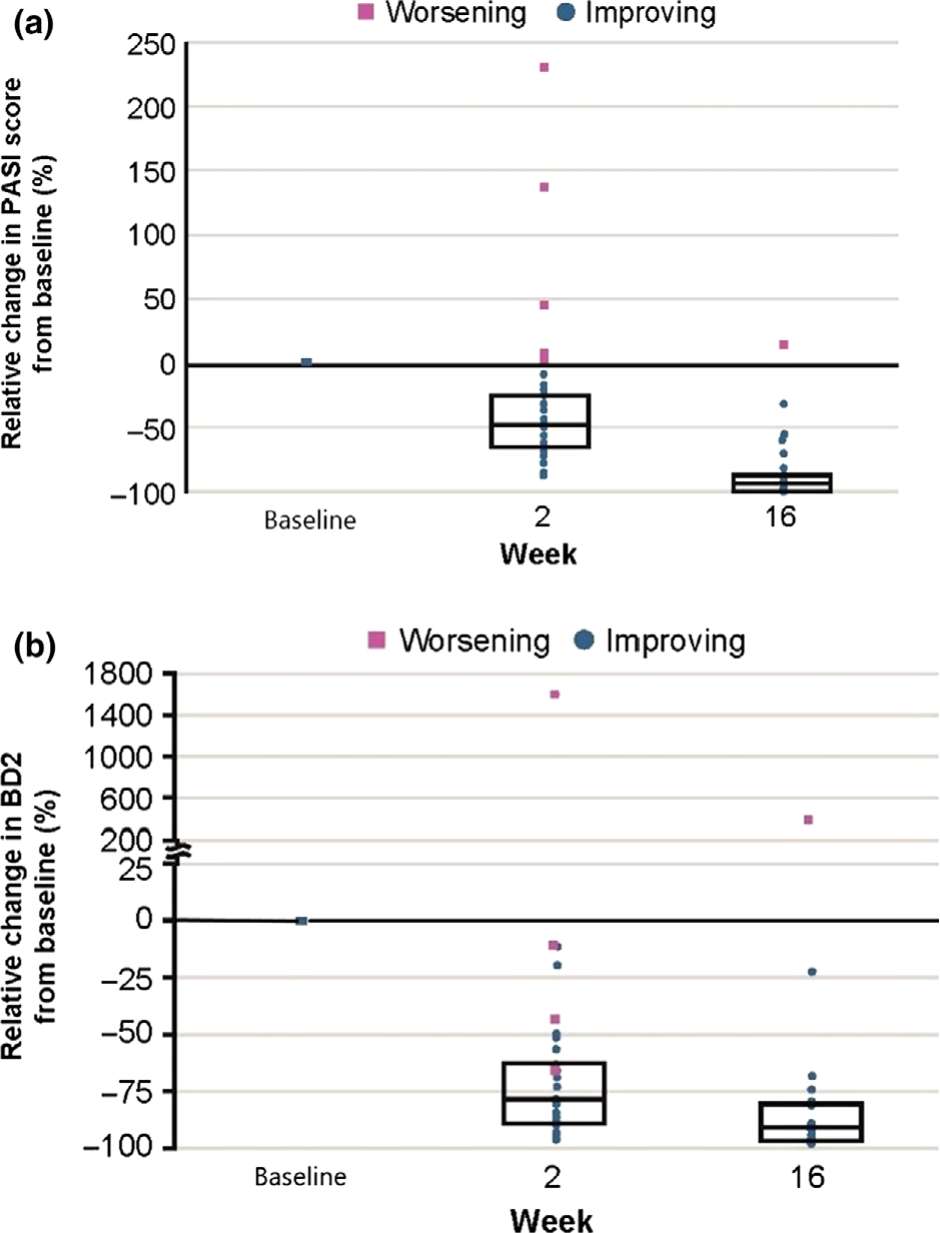
Relative change in individual PASI score from baseline and in individual serum BD‐2 level from baseline. PASI score and blood samples were collected at baseline, and weeks 2 and 16. Change of (a) PASI score and (b) serum BD‐2 at weeks 2 and 16 were evaluated relative to baseline. Data from FAS. BD, β‐defensin; FAS, full analysis set; PASI, Psoriasis Area and Severity Index.

The relative change in individual PASI score and serum BD‐2 level from baseline was plotted in Figure 3. At week 2, a remarkable decrease was observed in BD‐2 with a median percentage change of −79.5, and the decrease was greater than that of PASI (median percentage change of −47.7). Both BD‐2 and PASI levels further decreased at week 16 with median percentage change of −90.3 and −94.6, respectively. Of note, out of four patients whose PASI scores increased at week 2 from baseline (Fig. 3a), three patients exhibited a decrease in BD‐2 levels at the corresponding time point (Fig. 3b), and all of the patients subsequently showed decreased PASI scores at week 16. Inversely, one patient who retained an increased BD‐2 at week 2 continuously had an increased PASI up to week 16.

## Discussion

In the current study, treatment with secukinumab initiated immediately after discontinuing CyA led to an early improvement in PsO symptoms without CyA withdrawal‐associated relapse and was well‐tolerated without unexpected safety signals.

At baseline, when skin symptom was active even under CyA treatment, the median levels of IP‐10, TARC, IL‐16, IL‐17A, IL‐7, TNF‐α and VEGF‐A of the patients with PsO were over three‐times higher (394%, 988%, 374%, 371%, 2786%, 672% and 945%, respectively; Table 1) compared with the serum protein levels reported in healthy human subjects.^12^, ^13^, ^14^ Over three‐times lower protein levels were observed in IL‐1α and IL‐5 (8% and 24.7%, Table 1). The median baseline serum BD‐2 level among patients in this study was comparable with that of patients with active PsO (vs 5746 pg/mL [equivalent to ng/L], 102%) and was higher than the level in healthy volunteers (vs 82 pg/mL [equivalent to ng/L], 7134%) as previously reported by Kolbinger *et al.* 5

Among the proteins whose levels were elevated in patients with PsO at baseline compared with healthy human subjects, serum IL‐16 was correlated with disease activity. However, the levels of IL‐16 were relatively stable at weeks 2 and 16 when skin symptom was improved after the treatment with secukinumab. Increased IL‐17A levels were observed at weeks 2 and 16 despite the improved clinical disease activity at the corresponding time points. The increment of serum IL‐17A is likely attributable to the detection of the secukinumab‐IL‐17A complex that is eliminated slowly from circulation.^15^ As the current assay cannot distinguish between the free and secukinumab‐bound form of IL‐17A, it remains to be elucidated whether free IL‐17A level correlates with disease activity. Slight increase in serum TARC level was observed in week 2, which is in line with an earlier study.^3^ Serum IFN‐γ level at baseline in this study did not differ from the value obtained from healthy subjects (84%); however, IFN‐γ was negatively correlated with PASI at baseline and through the secukinumab treatment. The result appears to be in conflict with previous reports of a decreased mRNA expression in the PsO skin after IL‐17A inhibitor therapy,^16^ and a comparison of serum cytokine levels of patients with PsO to control subjects and an association of serum cytokines with PsO disease severity.^17^, ^18^ A possible influence of CyA on the cytokine/chemokine levels should be considered to interpret the difference.

β‐Defensin 2 is an antimicrobial peptide produced by epithelial cells including keratinocytes, the production of which is inducible by injury, infection or cytokines.^19^ In active psoriatic lesions, higher BD‐2 expression has been reported compared with the levels in atopic dermatitis and healthy skin.^20^ In the current study, there was a strong correlation between serum BD‐2 level and psoriatic disease activity, and the change in BD‐2 preceded the improvement in PASI score, suggesting that the suppression level of BD‐2 can predict the response to therapy. Further investigation measuring BD‐2 level at various conditions is needed to assess the possibility.

In addition to its biological antimicrobial function, BD‐2 is involved in the development of PsO by stimulating plasmacytoid dendritic cells and subsequent T‐cell activation.^21^, ^22^ In relation to the elevation of BD‐2 in psoriatic skin, Nograles *et al.* 23 demonstrated that human keratinocytes constitutively express IL‐17 receptors and that IL‐17A upregulated BD‐2 coding gene, *DEFB4*, in keratinocytes.

Moreover, *in vitro* data from human keratinocytes showed that IL‐17A induced *NFKBIZ*, which encodes a transcription co‐factor I kappa B‐zeta (IκB‐ζ), and that *NFKBIZ*/IκB‐ζ is required for the upregulation of IL‐17A‐induced genes including *DEFB4A*.^24^ *NFKBIZ* is rapidly induced by IL‐17A, but only to a lesser extent by TNF‐α stimulation in keratinocytes,^24^ and reduction in *DEFB4* mRNA level in lesional skin was larger after anti‐IL‐17A (ixekizumab) treatment compared with after anti‐TNF‐α (etanercept) in patients with PsO,^16^ suggesting that IL‐17A exclusively induces expression of IκB‐ζ, which subsequently activates the promoter of *DEFB4* for the production of BD‐2.

This study was conducted on a small sample size and included patients with PsO whose treatment was directly switched from CyA to secukinumab; hence, the serum protein levels of this population could have been under the influence of CyA.

Nonetheless, a rapid and robust reduction in serum BD‐2 was observed after secukinumab treatment in patients with PsO, change of which preceded the PASI score reduction. Serum BD‐2 is an easily measurable protein and could potentially be used as a specific surrogate biomarker for IL‐17A activity and to monitor responses to IL‐17A‐targeted therapies.

## Conflict of Interest

Y. T., K. M., M. Y. and R. T. are employees of Novartis Pharma K.K.. A. M. has received research grants, consulting fees and/or speaker’s fees from AbbVie, Boehringer Ingelheim, Celgene, Eli Lilly, Eisai, Janssen, Kyowa Hakko Kirin, LEO Pharma, Maruho, Mitsubishi Tanabe, Nichi‐Iko, Nippon Kayaku, Novartis, Sun Pharmaceutical Industries, Taiho Pharmaceutical and Torii Pharmaceutical. M. O. has received grants for research and/or honoraria for lectures and/or advisory board from Abbvie, Boehringer‐Ingelheim, Celgene, Eisai, Eli Lilly, Janssen, Kyowa Hakko Kirin, LEO Pharma, Maruho, Mitsubishi Tanabe Pharma, Novartis, Pfizer, Sanofi, Taiho Pharmaceutical and Torii Pharmaceutical.
